# Mosquito Species Associated Within Some Western Himalayas Phytogeographic Zones in the Garhwal Region of India

**DOI:** 10.1673/031.007.3201

**Published:** 2007-05-16

**Authors:** N. Pemola Devi, R.K. Jauhari

**Affiliations:** Parasitology Laboratory, Dept. of Zoology, D.A.V. (P.G.) College, Dehradun - 248 001, India

**Keywords:** immature, Culicines, Anophelines, breeding habitat similarity, cluster analysis

## Abstract

Thirty four species of mosquitoes (Diptera: Culicidae) were collected across three phytogeographic zones; tropical (300 to 1000 m), sub tropical (1000 to 2000 m) and temperate (2000 to 3000 m) in the Garhwal region of India. They included 5 genera: *Aedes, Anopheles, Armigeres, Culex* and *Uranotaenia*. Of these, the immature forms of 23 species were recovered from different breeding habitats. The larval habitats were seepage pools, river beds, rice fields, tanks, forest pools, ditches, streams, rock holes, tree holes, intradomestic containers and shallow pits. Three groups and two separate individual species were associated, based on breeding habitat similarity by means of cluster analysis. The characters taken into consideration for classification were natural/artificial, temporary/permanent, shady/lighted, vegetation, movement and turbidity. Breeding habitats such as streams and rock holes were the richest habitats shared by 18 mosquito species followed by seepage pools harboring 16 species of mosquitoes. The lowest species diversity (6 species) was recorded from shallow pits. Generally, all the collected species were found in natural habitats in quiet/stagnant conditions at a depth of 0.1–0.5 m. Generally, the maximum number of species preferred partially shady and temporary water habitats. Moderate vegetation and clear water habitats also had a diversity of mosquito species. *Culex mimeticus* Noe and *Anopheles maculatus* Theobald had the highest association coefficient (0.941) followed by *Anopheles stephensi* Liston and *Anopheles vagus* Donitz (0.884). The highest negative association (-0.30) was found between the species of *Culex vishnui* Theobald and *Culex brevipalpis* (Giles). There were a few species of mosquitoes for which only immatures were collected. Phytogeographically, the zones of lower elevation shared higher species abundance than the higher elevation.

## Introduction

Species never exist alone but always assemble together with other organisms to form a community in the same area or habitat. The co-existence of more than one species in a habitat at a given time indicates that species share habitat requirements. For example, different species of immature stages of mosquitoes occupy the same habitat and are, therefore, part of a guild. Understanding the relationship between habitats, environmental factors and occurrence of immature mosquitoes is essential for an efficient application of mosquito control methods. The majority of mosquito species preferred to breed in permanent breeding sites, especially natural sites in urban, semiurban and periurban areas, while some species are more abundant in temporary breeding places. Hence, the association between species of mosquitoes can provide clues to an understanding their biology and their role in the transmission of pathogens.

Senior White (1926) grouped the mosquitoes based on their preference of breeding habitats, which emphasized the ability of different species to select breeding habitats. Contributions made by earlier workers regarding breeding of different species under field conditions are entirely based on frequency of co-occurrence of the immature and have not been analysed statistically to know the strength of association or repulsion between them (Cole 1949; Bhat 1975a,b; Malhotra et al. 1987 and Bhatt et al. 1990). A number of studies have been carried out on Anopheline mosquito breeding in various habitats (Iyengar 1932; Russell and Rao 1940; Rahman et al. 1973; Rajagopalan et al. 1979; Sahu et al. 1990; Bhatt et al. 1993). The studies of Reisen et al. 1981, Savage *et al.* 1993, Almiron and Brewer (1996) and Rajnikant et al. 1998 demonstrate interspecific associations among mosquitoes and a correlation with physicochemical and biological composition of mosquito breeding waters. Further, Robert et al. 1998 studied the ecology of larval mosquitoes with special reference to *Anopheles arabiensis* Patton in market-garden wells in urban Dakar, Senegal. Subsequently, Minakawa et al. 1999 and Edillo et al. 2002 conducted studies on spatial distribution and habitat characterization of Anopheline mosquito larvae in western Kenya and West Africa respectively and discussed factors that influence their distribution patterns. Later on, Sattler et al. (2005) undertook studies on habitat characterization of *Anopheles* mosquito larvae in Dar es Salaam, Tanzania, and noted the turbidity and location of breeding habitats as key factors influencing the larval diversity. Rueda et al. 2005, 2006 described habitats and distribution patterns of *Anopheles sinensis* Wiedemann and the *An. hyrcanus* group. Recently, Aditya et al. 2006 conducted studies on larval habitats and temporal variations in mosquito diversity in the hill town of Darjeeling Himalayas, India, for a qualitative and quantitative assessment of mosquito distribution.

Some related mosquito faunal studies from the Garhwal region in the state of Uttaranchal, India, were made by Mahesh and Jauhari (2000a, 2003) and Pemola and Jauhari (2004 a, b), while studies on interspecific associations and the index of association among immature of Anophelines from the same region have been worked out by Jauhari et al. 1995, Mahesh et al. 1995 and Mahesh and Jauhari (2000 b).

During field surveys of mosquito breeding sites in the Garhwal region in the state of Uttaranchal, India, several environmental factors that could be quantified were found to be promising as predictors of mosquito occurrence (Pemola and Jauhari 2005). One of the most important factors was the vegetation that favors larval propagation and is correlated with adult densities. Moreover, habitat and climate has also been found to exhibit a correlation with the mosquito species that are present in an area.

Given that a thorough knowledge of physicochemical and biological parameters of the breeding habitats is essential, since each habitat produces specific mosquito species and shows a seasonal progression, we surveyed possible mosquito breeding sites in all three phytogeographic zones of Garhwal and grouped mosquito species associations according to the similarity of their chosen habitats. The present study is the first attempt to understand mosquito associations in different breeding habitats in the Garhwal region of India.

## Materials and Methods

### Study area

The survey was made between November 2000 to October 2002 from fixed localities across three phytogeographic zones in the Garhwal region: tropical (300–1000 m), sub tropical (1000–2000 m) and temperate (2000–3000 m). The study covered a major part of the 5 districts including Dehra Dun, Pauri, NewTehri, Chamoli and Uttarkashi located between 29°26′N to 31°26′N latitude and 77°39′E to 80°03′E longitude. As per earlier records, the western part of the Himalayas has been found to be extremely rich in plant life. Among the phytogeographic zones, the tropical zone is mainly covered by deciduous forest while sub-Himalayan region consists of sub-tropical forest type including pine forest and mixed oak and rhododendron vegetation. The third zone of temperate forest is composed of conifers, oak and rhododendrons. Details of the selected area such as longitude, latitude and altitude were computed using the global positioning system (GPS) for accurate location of sampling stations and the areas of interest. A portable altimeter was also used for measuring altitude of the mosquito's habitat.

**Table 1.**
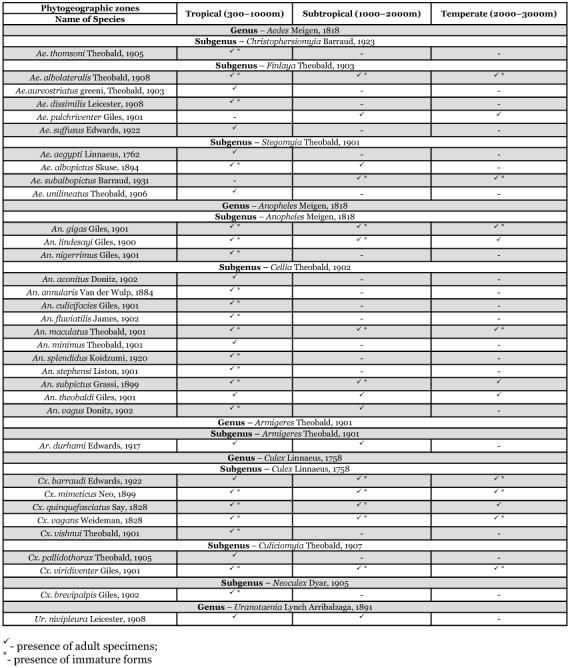
Distribution of Mosquitoes across the three different Phytogeographic zone in Garhwal region of Uttaranchal (India) during November 2000 to October 2002.

### Mosquito sampling

Adult mosquitoes were collected using aspirator and flash light from both indoor and outdoor resting habitats during morning hours (06:00–08:00) every two weeks. Collection of immature mosquitoes was also made on the same day by dipping and netting methods as per WHO (1975) guidelines. Identification is based on adult characters using standard taxonomic keys and catalogues (Christophers 1933; Barraud 1934; Wattal and Kalra 1961; Knight and Stone 1977; Das et al. 1990; Darsie and Pradhan 1990; Nagpal and Sharma 1995). Species were confirmed from adults that emerged in the laboratory from collected immatures. In each locality, the collection spots were fixed in different directions. In addition, random collection was done from every possible habitat. Information on co-existing biotic community and breeding characters was recorded at the time of mosquito sampling. Identified mosquito specimens are deposited in the insect collection records at Zoological Survey of India, Kolkata.

**Figure 1.**
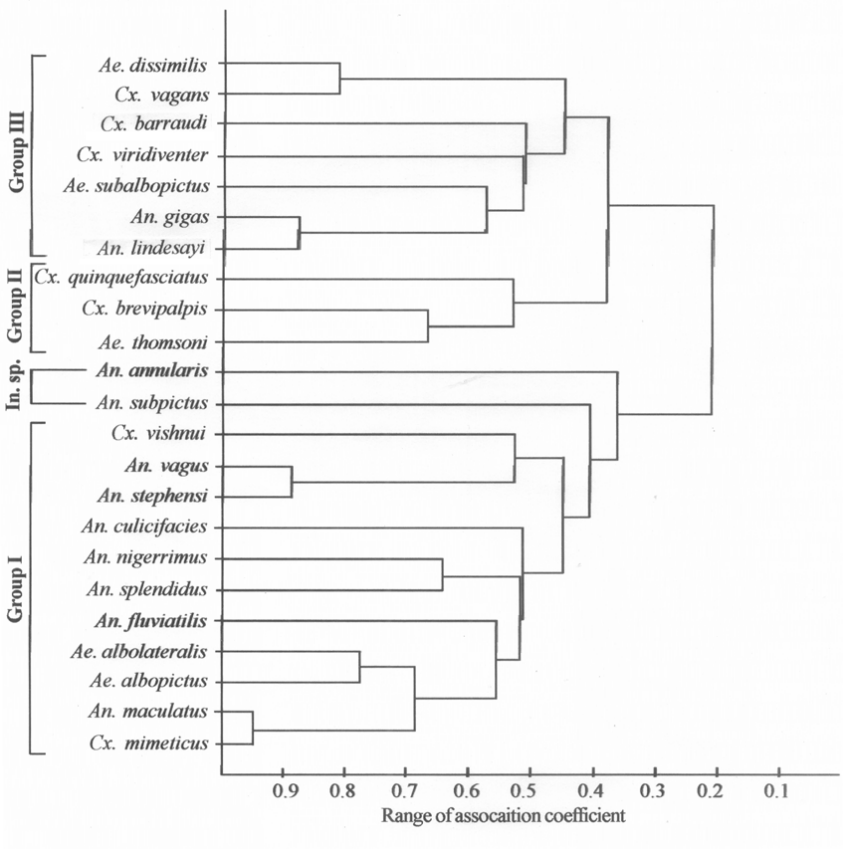
Phenogram of 23 OUT's resulting from Cluster analysis (based on Table 2).

### Data analysis

The collected data with respect of adult and immature mosquitoes were analyzed using the following steps - i) listing of operative taxonomic units, ii) development of basic matrix of data and iii) calculation of similarity for each pair of mosquito species. Since the main purpose was to group mosquito species with similar breeding features, the operative taxonomic units chosen were the mosquito species collected. The recorded breeding habitats and physical characters of breeding grounds were analyzed to identify common patterns of immature stage habitats where different mosquito species were collected. Both quantitative (water depth) and qualitative (natural / artificial, permanent / temporary, shady / lighted, water movement, vegetational condition and turbidity) characters were codified as 1/0 (= presence/absence).

**Table 2.**
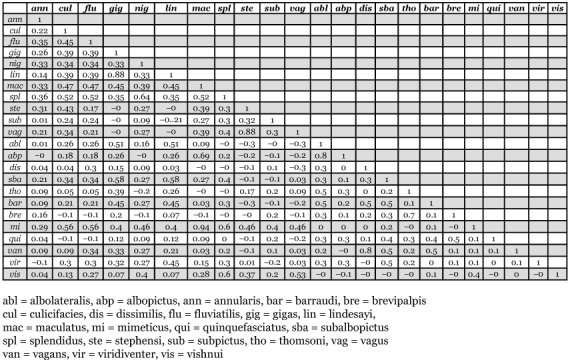
Similarity matrix for the operative taxonomic units calculated using coefficient of association.

A basic matrix of data was developed in tabular form on the basis of codified data, consisting of rows for mosquito species and columns for positive breeding habitats and characters. Values within the cells represent 1 or 0 if each character was recorded for each species or not, respectively. These data were used to analyze and calculate the similarity for all possible pairs of operative taxonomic units. The similarity for all possible combinations of mosquito species pairs (operative taxonomic units) was calculated using the coefficient of association and thereafter the similarity matrix was developed. Operative taxonomic units were grouped on the basis of similarity using cluster analysis (SAS Institute, 1987).

## Results

### Distribution of mosquito species

Altogether 34 species of mosquitoes within the following 5 genera, *Aedes, Anopheles, Armigeres, Culex* and *Uranotaenia*, were collected (Table 1). Among these, 23 species were immatures. Adults and immatures, 32 and 21 species respectively, were collected in the tropical zone at an elevation between 300–1000 m. 17 species of adults and 11 species of immatures were recovered from the subtropical zone, while from the temperate zone 13 and 8 species were found as adults and immatures respectively. There were a few species of mosquitoes for which only immatures were collected from a particular zone. Phytogeographically, the zones of lower elevation shared higher species abundance than the higher elevation.

### Grouping of immature mosquitoes

While compiling the data with respect of occurrence of immature vs. breeding characters, groups I, II and III were distinguished in the phenogram in addition to two individual species (Figure 1) according to the coefficient of association obtained (Table 2).

Group I included 11 species: *Culex mimeticus* Noe, *Anopheles maculatus* Theobald, *Aedes albopictus* Skuse, *Aedes albolateralis* Theobald, *Anopheles fluviatilis* James, *Anopheles splendidus* Koidzumi, *Anopheles nigerrimus* Giles, *Anopheles culicifacies* Giles, *Anopheles stephensi* Liston, *Anopheles vagus* Donitz, and *Culex vishnui* Theobald.

Group II included 3 species: *Aedes thomsoni* Theobald, *Culex brevipalpis* (Giles) and *Culex quinquefasciatus* Say.

Group III included 7 species: *Anopheles lindesayi* Giles, *Anopheles gigas* Giles, *Aedes subalbopictus* Barraud, *Culex viridiventer* Giles, *Culex barraudi* Edwards, *Culex vagans* Wiedemann and *Aedes dissimilis* Leicester.

**Table 3.**
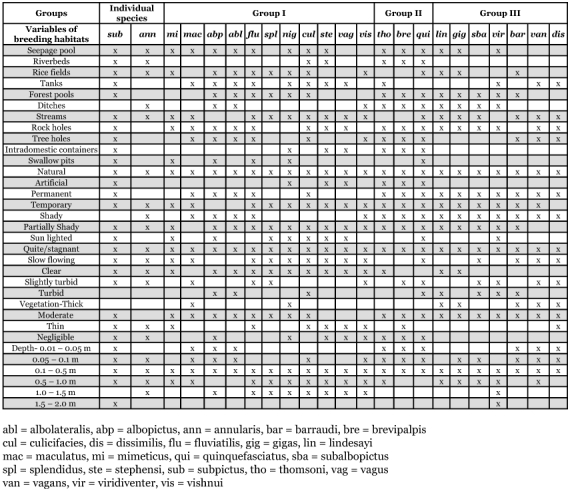
Showing mosquito breeding habitats/characters in respect of different groups of mosquitoes in Garhwal region of Uttaranchal (India) during November 2000 to October 2002.

Two species, *Anopheles annularis* Van der Wulp and *Anopheles subpictus* Grassi had unique preferences of habitat and association.

*Cx. mimeticus* and *An. maculatus* showed the highest association coefficient (0.941) followed by *An. stephensi* and *An. vagus* (0.884). There was a high positive association among the following mosquito associates: *An. gigas* with *An. lindesayi* (0.877) and *Ae. dissimilis* with *Cx. vagans* (0.819). Since the lowest coefficient of association (0.004) was found between *Cx. quinquefasciatus* and *Cx. viridiventer*, they were separated into groups II and III respectively. The highest negative association (-0.30) was found between *Cx. vishnui* and *Cx. brevipalpis* in groups I and II respectively (Table 2). Both *An. subpictus* and *An. annularis* showed close association with the species of group I but their coefficient of association was lower. The two species of *Aedes, Ae. albopictus* and *Ae. albolateralis*, had similar breeding preferences as compared to most of the Anopheline species. Among the *Culex* species, *Cx. mimeticus* and *Cx. vishnui* also showed similarity of habitat requirements with Anopheline species while in group III, *An. gigas* and *An. lindesayi* were found associated with Culicines (*Aedes* and *Culex* sp.) showing different habitat preference from other Anopheline species.

### Characters of breeding habitats

The positive breeding habitats, and their quantitative characters (water depth) and qualitative characters (natural/artificial, permanent/temporary, shady/lighted, water movement, vegetation condition and turbidity), are listed with regard to presence or absence of different mosquitoes species in Table 3. The breeding habitats of mosquitoes found in the present study were seepage pools, riverbeds, rice fields, tanks, forest pools, ditches, streams, rock holes, tree holes, intradomestic containers and shallow pits. Breeding habitats such as streams and rock holes were the richest habitats sharing 18 mosquito species followed by seepage pools harboring 16 species of mosquitoes. The lowest species diversity (6 species) was recorded from shallow pits. Generally, all the collected species were found in natural habitats in quiet/stagnant conditions at a depth of 0.1–0.5 m. In general, the maximum number of species preferred partially shady and temporary water habitats. Moderate vegetation and clear water habitats also had higher mosquito species diversity.

Mosquito species under Group I were mainly restricted to seepage pool, rice fields, tanks, stream and rock holes. Partially shady habitats, either slow flowing or quiet and clear to slightly turbid, were the main characters of the habitats. Moreover, the mosquito species mostly preferred the habitats located in the surroundings with moderate to thin vegetation. Immatures were also found in tree holes in which the water depth varied from 0.01–1.5 m. Seepage pool, riverbeds, forest pools, ditches, rock holes, tree holes and intradomestic containers were the common breeding habitats of the species of Group II. Shady to partially shady and quiet/stagnant habitats with moderate to negligible vegetation at a water depth of 0.1–0.5 m. were the main characters of the habitats preferred by Group II species. Group III species mostly preferred breeding habitats such as forest pools, streams and rock holes. All the species preferred natural breeding habitats of permanent conditions with shady, quiet/stagnant, moderate vegetation also at a water depth of 0.1–0.5 m. Temporary habitats with thick vegetation also harbored large numbers of species. The larvae of *An. subpictus* were found to breed in all of the above stated habitats except the ditches. The immature of *An. annularis* were found in seepage pools, riverbeds, rice fields, ditches, streams and tree holes. However, the natural habitats of temporary conditions with shady to partially shady habitats were the main characteristics of the habitats of this species.

## Discussion

Both quantitative and qualitative characters of the mosquito breeding habitats have contributed to understanding the similarity of habitat requirements of different species. Almiron and Brewer (1996) pointed out that different types of habitats, both natural and artificial, nature of vegetation, water movement and water depth are the main characters that explain the observed variations among mosquito species. Based on habitat similarity by means of cluster analysis, four groups of species have been associated but in the present study only 3 groups are recorded. The phenogram proposed by Almiron and Brewer (1996) with 19 operative taxonomic units, is different from that found in the present study that has 23 operative taxonomic units.

Bhat (1975a,b) studied the frequency of association of immatures of mosquitoes collected from the Himalayan range of India and recorded the highest association of *An. maculatus* with *Cx. mimeticus* which is similar to the present observation. Others, who have made related studies from parts of Garhwal are Pemola and Jauhari (2004b, 2005). Although there is some resemblance, their studies are based on distribution of mosquitoes in macro level features such as vegetation, altitudes and land use patterns. The findings of Bhatt et al. (1990, 1993) concentrated on the association among Anopheline immatures in different breeding habitats, that only slightly resembles the present study.

The present study has some similarities to the habitat requirements of most of the recorded mosquito species, especially *An. culicifacies* and *An. annularis* but from a different region (Sahu et al. 1990). There is little similarity with Gunasekaran et al. (1993) and Mahesh and Jauhari (2000b) as they recorded a positive association between *An. culicifacies* and *An. annularis*, however in the present study a positive correlation between *An. culicifacies, An. aconitus* Donitz, *An. stephensi* and *An. subpictus* has been recorded. On other hand, Jauhari et al. 1995 observed associations of *An. culicifacies* and *An. fluviatilis* with *Aedes aegypti* (Linnaeus) and *Cx. quinquefasciatus* in the forested areas of Doon Valley, but in our findings the association was not the same as *An. culicifacies* and *An. fluviatilis* showed negative association with *Cx. quinquefasciatus* (i.e. -0.05) and larvae of *Ae. aegypti* were rarely found and hence not considered.

There are a number of papers on the relationship between vegetation and immature stages by several authors (Savage et al. 1990; Rajmankova et al. 1991, 1992; Rodriguez et al. 1993; Rajnikant et al. 1996) and almost all of them reported that larval abundance is related to the presence of a particular kind of vegetation. Their results get support from Aditya et al. 2006 who found cemented temporary pools containing maximum food resources, in term of detritus, vegetation and algae allowing the maximum number of species of different guilds to coexist.

In the present study, mosquito immatures found in turbid water were almost always Culicines, which is similar to the findings of Sattler et al. (2005). The preference of the immatures of Anophelines to breed in clear to slightly turbid water is similar to the findings of Bates (1994) and Robert et al. 1998. However, Gimning et al. 2001 found increasing *An. gambiae* s.l. larval densities with increasing turbidity. Further, the results of the present findings are contrary to those of Minakawa et al. (1999) and Edillo et al. 2002 in having different mosquito species as well as fluctuating ecological conditions prevailing in the area.

Considering the results of the present study in comparison to earlier findings, it has been found that positive associations between mosquito species may result from a common preference for a particular habitat, while negative associations may cause variation in preference for a particular habitat. Moreover, on the basis of associations observed between Anophelines in different habitats, it is also possible to group them as a distinct community. Maximum immature associations, as recorded in the habitats such as streams, rockholes and seepage pools, suggest high survival rate, ovipositional preferences and favorable physicochemical characteristics of these habitats. It was also noticed that prolonged water logging with fast changing ecological conditions and extensive surface area of habitats offer favorable breeding conditions to a number of mosquito species including disease vectors. The co-existence of more than one species in a habitat at a given time indicates that mosquito species of the same nature and preference interact with each other. In order to better elucidate the association between larval occurrence and abundance and environmental variables, further research should be made to examine more variables, including a detailed analysis of water chemistry and the ecology of mosquito predators.

## References

[bibr01] Aditya G Pramanik MK Saha GK 2006 Larval habitats and species composition of mosquitoes in Darjeeling Himalayas, India. *Journal of Vector Borne Diseases* 43 7 15 16642780

[bibr02] Almiron WR Brewer ME 1996 Classification of immature stage habitats of Culicidae (Diptera) collected in Cordoba, Argentina. *Memorias do Institute Oswaldo Cruz, Rio de Janeiro* 91 1 9 10.1590/s0074-02761996000100001 8734943

[bibr03] Barraud PJ 1934 *The fauna of British India, Ceylon and Burma. Diptera Vol. V, Family Culicidae, Tribe Megarhinini and Culicini*. 463 Taylor and Francis

[bibr04] Bates M 1949 *The natural history of mosquitoes*. The Macmillan Company

[bibr05] Bhat HR 1975a A survey of haematophagous arthropods in Western Himalayas, Sikkim and hill districts of West Bengal : Records of mosquitoes collected from Himalayan region of West Bengal ad Sikkim with ecological notes. *Indian Journal of Medical Research* 63 232 241 2554

[bibr06] Bhat HR 1975b A survey of haematophagous arthropods in Western Himalayas, Sikkim and hill districts of West Bengal : Records of mosquitoes collected from Himalayan region of Uttar Pradesh with ecological notes. *Indian Journal of Medical Research* 63 1584 1608 4372

[bibr07] Bhatt RM Sharma RC Kohli VK 1990 Interspecific associations among Anophelines in different breeding habitats of Kheda district, Gujarat Part I: Canal irrigated area. *Indian Journal of Malariology* 27 167 172 2292321

[bibr08] Bhatt RM Sharma RC Srivastava HC Gautam AS Gupta DK 1993 Interspecific associations among Anophelines in different breeding habitats of Kheda district Gujarat: Part II-Non-Canal area. *Indian Journal of Malariology* 30 91 100 8405599

[bibr09] Christophers SR 1933 The fauna of British India including Ceylon and Burma. Diptera, Vol. IV, Family Culicidae, Tribe Anopheline Taylor and Francis London 371

[bibr10] Cole LC 1949 Measurement of Interspecific association. *Ecology* 30 411 424

[bibr11] Darsie RF Pradhan SP 1990 The mosquitoes of Nepal: Their Identification and Biology. *Mosquito Systematics* 22 69 130

[bibr12] Das BP Rajagopal R Akiyama J 1990 Pictorial keys to the species of Indian Anophelines mosquitoes. *Zoology* 2 131 162

[bibr13] Edillo FE Toure YT Lanzaro GC Dolo G Taylor CE 2002 Spatial and habitat distribution of *Anopheles gambiae* and *Anopheles arabiensis* (Diptera: Culicidae) in Banambani Village, Mali. *Journal of Medical Entomology* 39 70 77 11931274 10.1603/0022-2585-39.1.70

[bibr14] Gimning JE Ombok M Kamau L Havlett WA 2001 Characteristics of larval Anopheline (Diptera: Culicidae) habitats in Western Kenya. *Journal of Medical Entomology* 38 282 288 11296836 10.1603/0022-2585-38.2.282

[bibr15] Gunasekaran K Jambulingam P Das PK 1993 Interspecific association and Index of Association of *Anopheles fluviatilis* James in the breeding habitats in hill tract of Koraput district, Orissa. *Journal of Communicable Diseases* 25 156 163

[bibr16] Iyengar MOT 1932 *Anopheles* breeding in relation to season. *Indian Journal of Medical Research* 19 917 939

[bibr17] Jauhari RK Singh RP Singh S 1995 Breeding and resting site of mosquitoes in the forested areas of Doon Valley. *Indian Journal of Forestry* 18 249 250

[bibr18] Knight KL Stone A 1977 A catalogue of the mosquitoes of the World (Diptera: Culicidae). 611 The Thomas Say Foundation Entomological Society of America

[bibr19] Mahesh RK Jauhari RK 2000a Resurgence of sylvatic mosquitoes in Doon Valley. *Journal of Parasitic Diseases* 24 147 150

[bibr20] Mahesh RK Jauhari RK 2000b Interspecific association and index of association among different aquatic forms of Anophelines collected from Saharanpur block of Doon Valley. *Journal of Parasitic Diseases* 24 147 150

[bibr21] Mahesh RK Jauhari RK 2003 Mosquito fauna of the forested areas of Doon Valley,(UP) India. *Entomon* 28 185 190

[bibr22] Mahesh RK Singh RP Singh S Jauhari RK 1995 Breeding of mosquitoes in urban areas of city Dehradun. *Uttar Pradesh Journal of Zoology* 15 100 102

[bibr23] Malhotra PR Sarkar PK Das NG Hazarika S John VM 1987 Mosquito survey in Tirap and Subansiri districts of Arunachal Pradesh. *Indian Journal of Malariology* 24 151 158 2898389

[bibr24] Minakawa N Mutero CM Githure JI Beier JC Yan G 1999 Spatial distribution and habitat characterization of Anopheline mosquito larvae in Western Kenya. *American Journal of Tropical Medicine and Hygiene* 61 1010 1016 10674687 10.4269/ajtmh.1999.61.1010

[bibr25] Nagpal BN Sharma VP 1995 *Indian Anophelines*. Oxford and IBH Publishing Co. Pvt. Ltd

[bibr26] Pemola Devi N Jauhari RK 2004a Mosquito records from Garhwal region (Uttaranchal). *Journal of Experimental Zoology, India* 7 237 244

[bibr27] Pemola Devi N Jauhari RK 2004b Altitudinal distribution of mosquitoes in mountainous areas of Garhwal region Part — I. *Journal of Vector Borne Diseases* 41 17 26 15332482

[bibr28] Pemola Devi N Jauhari RK 2005 Species diversity patterns among mosquitoes (Diptera: Culicidae) from certain parts in Garhwal Himalayas, India. *Journal of Applied Bioscience* 31 105 113

[bibr29] Rahman SJ Wattal BL Sharma MID 1973 Ecology of mosquitoes of Nilgiri Hills (Tamil Nadu) with particular reference to vectors of human diseases. *Indian Journal of Entomology* 35 228 246

[bibr30] Rajagopalan PK Chandrahas RK Paniker KN 1979 Mosquito selection in Pattu and different adjacent localities in Thanjavur district (Tamil Nadu) with particular reference to *Anopheles culicifacies* Giles. *Indian Journal of Medical Research* 69 589 597 457199

[bibr31] Rajmankova E Savage HM Rodriguez MH Roberts DR Rejmanek M 1992 Aquatic vegetation as a basis of classification of *Anopheles albimanus* Weideman (Diptera: Culicidae) larval habitats. *Environmental Entomolog* 21 598 603

[bibr32] Rajmankova E Savage HM Rejmanek M Arredondo-Jimenez JI Roberts DR 1991 Multivariate analysis of relationships between habitats, environmental factors and occurrence of Anopheline mosquito larvae *Anopheles albimanus* and *An.* 311*pseudopunctipennis* in Southern Chiapas, Mexico. *Journal of Applied Ecology* 28 827 841

[bibr33] Rajnikant Pandey SD Sharma SK 1996 Role of biological agents for the control of mosquito breeding in rice fields. *Indian Journal of Malariology* 33 209 215 9125835

[bibr34] Rajnikant Pandey SD Sharma SK Sharma VP 1998 Species diversity and Interspecific associations among mosquitoes in rice Agro-ecosystem of Kheda district, Gujarat. *Indian Journal of Malariology* 35 22 30 10319558

[bibr35] Reisen WK Siddiqui Aslamkhan M Malik GM 1981 Larval interspecific associations and physicochemical relationship of the ground water breeding mosquitoes of Lahore, Pakistan. *Journal of Scientific Research* 33 1 23

[bibr36] Robert V Awono-Ambene HP Thioulouse J 1998 Ecology of larval mosquitoes, with special reference to *Anopheles arabiensis* (Diptera: Culicidae) in Market-Garden Wells in Urban Dakar, Senegal. *Journal of Medical Entomology* 35 948 955 9835685 10.1093/jmedent/35.6.948

[bibr37] Rodriguez AD Rodriguez MH Meza RA Hernandez JE Rejmankova E Savage HM Roberts DR Pope KO Legters L 1993 Dynamics of population densities and vegetation associations *of Anopheles albimanus* larvae in a coastal area of southern Chiapas, Mexico. *Journal of American Mosquito Control Association* 9 46 58 8468574

[bibr38] Rueda LM Iwakami M O'Guinn M Mogi M Prendergast BF Miyagi I Toma T Pecor JE Wilkerson RC 2005 Habitats and distribution of *Anopheles sinensis* and associated *Anopheles hyrcanus* Group in Japan. *Journal of American Mosquito Control Association* 21 458 463 10.2987/8756-971X(2006)21[458:HADOAS]2.0.CO;2 16506573

[bibr39] Rueda LM Kim HC Klein TA Pecor JE Li C Sithiprasasna R Debboun M Wilkerson RC 2006 Distribution and larval habitat characteristics of *Anopheles hyrcanus* Group and related mosquito species (Diptera: Culicidae) in South Korea. *Journal of Vector Ecology* 31 199 206 10.3376/1081-1710(2006)31[198:dalhco]2.0.co;2 16859110

[bibr40] Russell PF Rao HR 1940 On habitat and association of species of Anopheline larvae in south-eastern Madras. *Journal of Malarial Institute of India* 3 153 178

[bibr41] Sahu SS Parida SK Sadanandane C Gunasekaran K Jambulingam P Das PK 1990 Breeding habitats of malaria vectors: *An. fluviatilis, An. annularis* and *An. culicifacies* in Koraput district, Orissa. *Indian Journal of Malariology* 27 209 216 2093004

[bibr42] SAS Institute 1987 SAS *user's guide: SAS/STAT*, version 6 SAS Institute, Inc. Cary, North Carolina

[bibr43] Sattler MA Mtasiwa D Kiama M Premji Z Tanner M Killeen GF Lengeler C 2005 Habitat characterization and spatial distribution of *Anopheles sp.* Mosquito larvae in Dar es Salaam (Tanzania) during an extended dry period. *Malaria Journal* 4:4, http://www.malariajournal.com/content/4/1/4. 10.1186/1475-2875-4-4 PMC546229 15649333

[bibr44] Savage HM Rejmankova E Arredondo-Jimenez JI Roberts DR Rodriguez MH 1990 Limnological characterization of larval habitats for two primary malarial vectors, *Anopheles albimanus* and *Anopheles pseudopunctipennis*, in coastal areas of Chiapas State, Mexico. *Journal of American Mosquito Control Association* 6: 612–620. Senior White R. 1926. Physical factors in mosquito ecology. *Bulletin of Entomological Research* 16 187–248. 339–248 2098467

[bibr45] Wattal BL Kalra NL 1961 Regionwise pictorial keys to the female Indian Anopheles. *Bulletin of National Society of India for Malaria and other Mosquitoborne Disease* 9 85 138

[bibr46] WHO 1975 *Manual on practical entomology in malaria vector bionomics and organization of antimalaria activities.* Part I and part II, Offset Publication, No. 13 Geneva

